# Consumer misconceptions and socioeconomic determinants of broiler meat consumption in Bangladesh

**DOI:** 10.5455/javar.2026.m1042

**Published:** 2026-06-22

**Authors:** Seikh Masudur Rahman, M. Wakilur Rahman, Md Mahfuzul Hasan, Most. Shahana Akter, Rokshana Parvin, Emdadul Haque Chowdhury

**Affiliations:** 1 Department of Pathology, Faculty of Veterinary Science, Bangladesh Agricultural University, Mymensingh 2202, Bangladesh; 2 Department of Rural Sociology, Faculty of Agricultural Economics and Rural Sociology, Bangladesh Agricultural University, Mymensingh 2202, Bangladesh; 3 Department of Agribusiness and Marketing, Faculty of Agricultural Economics and Rural Sociology, Bangladesh Agricultural University, Mymensingh 2202, Bangladesh

**Keywords:** Broiler meat, misconception, antibiotic residues, consumer preference, OLS

## Abstract

**Objectives:** The poultry industry makes a significant contribution to food security, employment generation, and affordable protein supply in Bangladesh. This study aims to investigate consumer misconceptions about broiler meat and to identify the socioeconomic factors influencing consumer preferences.

**Materials and Methods:** Survey data were collected from 369 consumers across rural, semi-urban, and urban areas. Respondents represented diverse occupational groups, including farmers, students, businessmen, and service holders. Descriptive statistics and econometric analyses (Ordinary Least Squares method) were applied. Misconceptions were measured using a five-point Likert scale, ranked through index values.

**Results:** Consumers exhibited strong concerns about hygiene at processing points and associated broiler meat with unsafe chemicals, growth hormones (4.2), heavy metals (4.1), and lower nutrition compared to Deshi meat (4.06). Respondents even perceived that broiler meat might cause cancer. Ordinary Least Squares (OLS) estimation shows that residence, education, occupation, and income significantly influenced broiler meat consumption. Urban residence and higher education were positively associated with consumption, whereas higher income and professional occupations showed a negative relationship.

**Conclusions:** The study provides new evidence on how perceptions and socioeconomic factors interact to shape broiler meat consumption in Bangladesh, offering insights for nutritional policy remodeling and poultry sector sustainability. It also highlights the importance of addressing consumer misconceptions and strengthening food safety communication to sustain broiler meat consumption all over Bangladesh.

## 1. Introduction

The global poultry industry is among the most rapidly expanding sectors over the last few decades due to its affordability, efficient protein conversion rate, and advances in farm technology [1]. Progress from farming to processing has played a pivotal role in propelling poultry into one of the fastest-expanding segments of the world livestock industry. The global poultry market is projected to expand by approximately 2.5% to 3%, especially in the emerging regions such as Asia, Latin America, the Middle East, and Africa [2]. The developed world of Europe continues to grow, albeit at a slower rate. Despite these encouraging trends, the sector faces significant threats like avian influenza outbreaks, rising feed costs with global warming, and geopolitical tensions that could disrupt trade flows. Nevertheless, continued investment, innovation, and supply-demand strategy management are key to sustaining long-term growth in the poultry sector. This industry plays a significant role in economic development, food security, and rural livelihoods across various regions. Investments in the poultry industry led to modernization through advanced breeding techniques, improved feed, and automation, which enhance production efficiency and reduce losses [3]. In addition, effective supply chain management and adaptability to unforeseen events are essential for business agility in the poultry industry [4].

In this global context, Bangladesh’s poultry industry has been transformed rapidly from traditional backyard farming to a commercially intensive sector since the early 2000s. This transition has contributed significantly to employment generation, income creation, and the supply of affordable animal protein [5, 6]. Broiler meat currently accounts for approximately 37% of total animal protein consumption in the country [7]. Rapid urbanization, rising incomes, and changing dietary patterns have further accelerated sectoral growth. Subsidies by the government, tax exemptions, and the adoption of advanced farming technologies have further supported sectoral development. This is evidenced by the massive international interest and investment witnessed in events such as the 13th International Poultry Show and the WPSA-BB Fair 2025, where business transactions amounting to thousands of crore taka were negotiated, and green technology and environmentally friendly endeavors espoused. The impacts of the COVID-19 pandemic, cost inflation, changed consumer behavior, and continuous healthcare concerns have slowed growth [8].

Although broiler meat continues to be the best affordable option, consumer preferences have increasingly shifted towards local chicken due to its supposed improved flavor, texture, and nutrient content. It is also driven by the impression that broiler chickens are produced too fast through chemicals and raised in unclean or unsafe conditions [9, 10]. Experts in the industry emphasized farm registration, waste management, recycling, and green technology like solar power for net-zero carbon targets. Biogas production from poultry waste not only generates energy but also contributes to a circular economy within the industry [11]. Plans are also in motion to enhance farmer training, expand small farms, and ensure equitable protein distribution. The occasion was a testament to Bangladesh’s promise as a poultry model for South Asia, with continued international collaboration, especially with the Netherlands, having a significant role to play in its future development.

A significant portion of the Bangladeshi population holds persistent misconceptions about broiler meat. Common beliefs include the presence of harmful chemicals, antibiotics, heavy metals, and even cancer-causing agents [12, 13, 14, 15]. Concerns over bacterial contamination, particularly *E. coli* and *Salmonella*, have further contributed to consumer mistrust. Broiler meat is also perceived as inferior to local meat, often attributed to visible defects like wooden breast and white striping [16]. These perceptions persist despite evidence that broiler meat, when produced under hygienic and regulated conditions, can be both safe and nutritious [17, 18]. Lower-income groups often consume conventional broiler meat, which is perceived as less safe, while higher-income consumers are willing to pay a premium for safer options [14].

These misconceptions shape what people choose to eat. In Bangladesh, meat intake is still quite low, about 4.4 kg per person each year based on the World Population Review [19]. So, dealing with these concerns matters more than ever. While studies have examined general consumer preferences for chicken meat [20], few have focused specifically on the socio-economic and perceptual factors influencing broiler meat consumption in Bangladesh [14, 21, 22]. Research indicates that consumers exhibit a strong preference for safe broiler chicken meat, driven by heightened awareness of health risks associated with conventional poultry production methods [14]. The pandemic has intensified consumer focus on food safety, leading to shifts in purchasing behavior and preferences for quality over price.

Despite existing studies on poultry consumption, limited research has jointly examined consumer misconceptions and socioeconomic determinants within a unified empirical framework in Bangladesh. This study contributes by integrating perception analysis with econometric modeling to provide a more comprehensive understanding.

## 2. Materials and Methods

### 2.1. Ethical approval

The ethical committee of the Bangladesh Agricultural University Research System has approved this study. The approval number is BAURES/PR/44/ESRC/ECON2025.

### 2.2. Conceptual framework of broiler meat consumption

The conceptual framework for broiler meat consumption in Bangladesh is developed based on the consumer behavior theory [23], the Theory of Planned Behavior (TPB) [24], and the Diffusion of Innovation Theory [25]. These theories are operationalized using observable survey variables that are later incorporated into the econometric model (Table 5).

**Table 5.** The factors affecting broiler meat consumption.

Variable	Category	Coefficient	Std. err.	t	*p* > t
Location (Base: Rural)	Semi-urban	1.76	0.18	9.54	**0.00**
	Urban	2.37	0.21	11.08	**0.00**
Gender (Male)		0.35	0.28	1.24	0.22
Total family members		–0.04	0.04	–0.96	0.34
Age		0.00	0.01	0.18	0.86
Education (Base: No Education)	Primary	0.62	0.46	1.34	0.18
	Up to Secondary	0.84	0.47	1.78	**0.08**
	Up to tertiary	0.95	0.48	1.96	**0.05**
Occupation (Base: Farmers)	Businessman	–1.75	0.27	–6.51	**0.00**
	Service holder	–1.14	0.22	–5.27	**0.00**
	Housewife	0.66	0.48	1.39	0.17
	Others	0.07	0.25	0.28	0.78
Monthly Income		–0.00	0.00	–3.02	**0.00**
Monthly Expenditure		0.00	0.00	–0.23	0.81
_cons		13.06	0.75	17.47	0.00

**Note:** Prob > F = 0.0000; R-squared = 0.7495; Adj. R-squared = 0.7396; Root MSE = 1.5131.

Consumer behavior theory explains how individual characteristics influence food choices. To identify personal and demographic influences on consumption behavior, this study considered age, gender, education, and occupation. It is assumed that younger consumers are more likely to adopt fast-food consumption habits and prefer broiler meat for convenience, whereas older consumers tend to be more health-conscious and therefore prefer to consume less. Similarly, education and occupation shape perceptions of health, nutrition, and convenience, reinforcing how personal attributes affect food choices [26].

The Theory of Planned Behavior (TPB) is applied by linking its core constructs to measurable proxy variables used in the regression model. Attitude toward behavior is represented by consumer perceptions and beliefs regarding broiler meat. Although attitudinal variables were primarily analyzed using Likert-scale perception indices (Table 4), their influence is indirectly reflected in consumption behavior (dependent variable in Table 5). Subjective norms use indirect proxies for social influence, including occupation and household structure (family size), which reflect social influence, peer behavior, and family-level decision-making processes. For example, occupational groups (e.g., farmers, service holders, businessmen) may exhibit different consumption norms shaped by social networks and professional environments. In addition, economic and access-related variables are considered for measuring Perceived Behavioral Control (PBC). Monthly income, monthly expenditure, education level, and place of residence (rural, semi-urban, or urban) are considered in this part. Residential location (rural, semi-urban, or urban) is interpreted through the diffusion of innovation theory. Urban consumers are more likely to adopt broiler meat consumption earlier due to better infrastructure, market access, and exposure to modern food systems. In contrast, rural consumers may adopt more gradually due to stronger attachment to traditional food sources. These variables capture consumers’ financial capacity, access to markets, and ability to purchase broiler meat. On the other hand, broiler meat consumption (frequency per week) is taken as a dependent variable.

**Figure 1.**
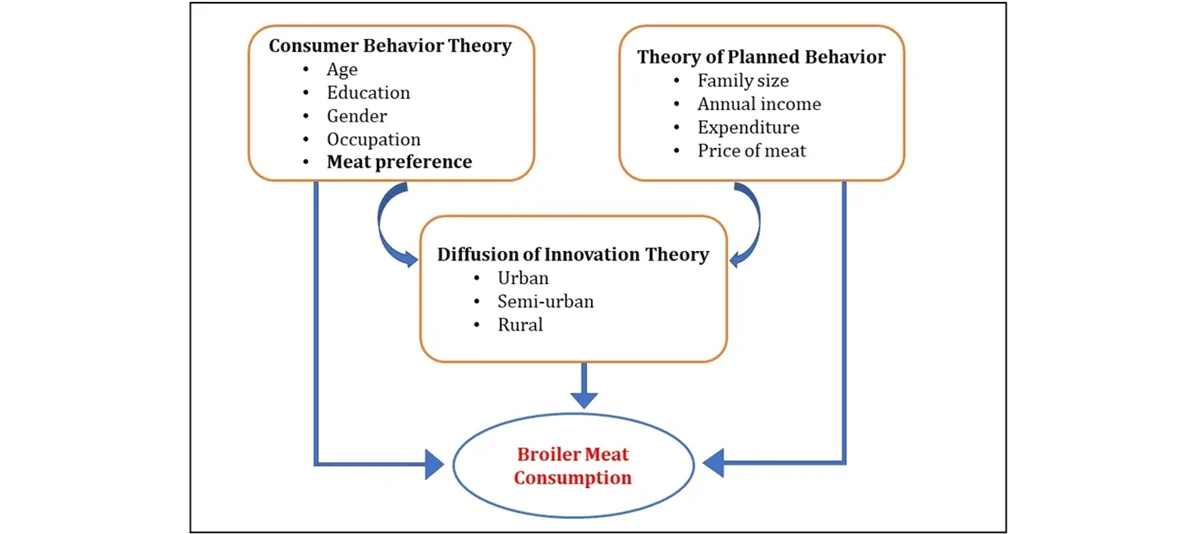
Operationalization of the conceptual framework for research incorporating theoretical understandings.

The amalgamation of consumer behavior theory, the theory of planned behavior, and diffusion of innovation theory establishes a robust framework for analyzing disparities in grilled meat consumption among Bangladeshi households (Figure 1).

### 2.3. Sources of data

A structured questionnaire was developed to collect primary data from 369 respondents across 12 districts of Bangladesh, covering rural (48.2%), semi-urban (32.0%), and urban (19.8%) areas (Table 1). The people who answered were farmers, businesspeople, students, and people who worked in services. Using Kobo Collect software, digital devices were used to collect the data. The questionnaire was tested ahead of time to make sure it was clear and reliable.

The survey was done in Bengali so that everyone could understand it. For respondents with limited literacy, trained enumerators assisted in administering the questionnaire. Data were analyzed using statistical software (STATA). Secondary data used in this study were obtained from FAO, the World Population Review database, and other published sources.

### 2.4. Analytical techniques

Descriptive analysis was conducted to unveil the socio-demographic profiles of the respondents and their attitudes towards consuming broiler meat in Bangladesh. Statistical measures such as frequency, mean, standard deviation, maximum, and minimum were calculated where applicable. Since some of the variables were categorical in nature and ranged from 1 to 5, descriptive statistics were also calculated to determine the percentage distribution of each response category. Additionally, an index-based ranking method was employed to analyze and rank respondents’ perceptions. A hierarchical ranking of pre-decided perception indicators was used to identify the primary reasons for rejecting broiler meat.

Econometric analysis was also undertaken using regression (OLS) techniques, in which the consumption of broiler meat was considered the dependent variable. The dependent variable was the self-reported frequency of consumption per week, considering a continuous scale for regression analysis. Higher values indicate more frequent consumption. In addition, location, age, gender, family size, number of children, education level, income, and expenditure were the independent variables. The OLS model was selected due to the continuous nature of the dependent variable (consumption frequency) and its suitability for estimating linear relationships between socioeconomic factors and consumption behavior.


\[
\begin{split}
y\; =  \;\alpha \;{\mathrm{ + }}\;\beta 1Location\;{\mathrm{ + }}\;\beta 2Gender\; + \;\beta 3total\;member\;{\mathrm{ + }}\;\beta 4Age\;{\mathrm{ + }}\;\beta 5Education\\
\;{\mathrm{ + }}\;\beta 6Occupation\;{\mathrm{ + }}\;\beta 7Income\;{\mathrm{ + }}\;\beta 8Expenditure
\end{split}
\]

Where: *y* = frequency of broiler meat consumption; *α* = intercept; *β*1 – *β*8 = coefficients of explanatory variables.

## 3. Results and Discussion

### 3.1. Status of the poultry sector

Table 2 demonstrates a steady upward trend in production, domestic supply, and food supply values between 2015 and 2022. Poultry production rose slowly from 236 to 284 thousand tons, showing that output improved slightly over the time period. Domestic supply followed the production trend in the year 2019. In terms of nutritional availability, the total food supply rose markedly from approximately 361,000 million kilocalories in 2015 to over 416,000 million kilocalories in 2022. This growth translated into an increase in daily per capita calorie availability from 6.3–6.7 kcal per capita per day from poultry sources. Although the rise appears moderate, it reflects a consistent enhancement in national food security and nutritional accessibility. These findings, drawn from the FAO [27] dataset, highlight that domestic food availability is improving.

**Figure 2.**
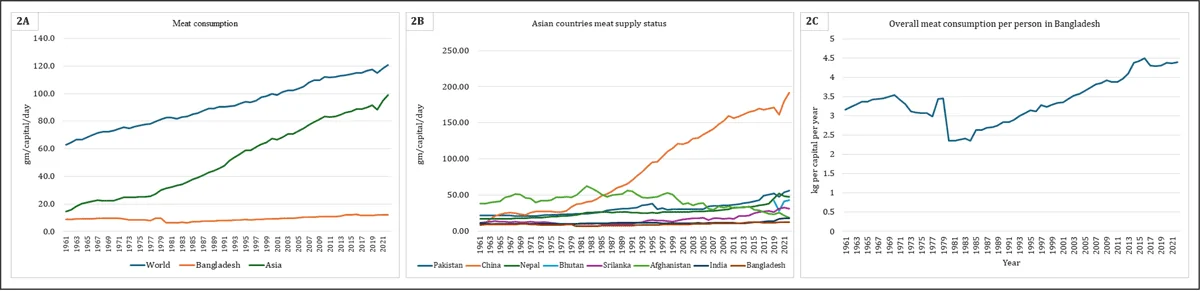
A) Meat consumption per capital per day. B) Asian countries’ meat supply status per day. C) Overall meat consumption per person (kg per year per capital).

Figure 2A compares per capita meat consumption in a day in Bangladesh with Asia and the world average over time. While global and Asian averages have shown a steady upward trend, Bangladesh’s consumption has remained almost static. China demonstrates a sharp rise in meat supply, driven by rapid economic growth, urbanization, and changing dietary preferences, while Afghanistan exhibits a declining trend, possibly due to economic and political instability (Figure 2B). Bangladesh, by contrast, occupies a lower position but shows a gradual increase since 1961, albeit at a much slower pace compared to regional peers. Figure 2C further shows that Bangladesh’s per capita meat consumption per year was only 4.4 kg in 2022, which is considerably lower than regional and global averages. Overall, the results indicate that while global and Asian meat consumption has increased steadily, Bangladesh has experienced only marginal growth. This highlights a persistent gap in dietary protein intake despite economic progress.

**Table 1.** Distribution of surveyed population by category and regions.

Category	Rural	Semi-urban	Urban	All
Farmer	52 (29.21%)	33 (27.97%)	24 (32.88%)	109 (29.54%)
Businessman	41 (23.03%)	31 (26.27%)	11 (15.07%)	83 (22.49%)
Service holder	45 (25.28%)	28 (23.73%)	23 (31.51%)	96 (26.02%)
Housewife	7 (3.93%)	9 (7.63%)	4 (5.48%)	20 (5.42%)
Others	33 (18.54%)	17 (14.41%)	11 (15.07%)	61 (16.53%)
Total	178(48.2%)	118(32.0%)	73(19.8%)	369(100%)

**Table 2.** Temporal changes in poultry production, supply, and per capita food energy contribution in Bangladesh (2015–2022).

Year	Production (1000 Tons)	Domestic supply quantity	Export quantity	Total Food Supply (million kcal/year)	Per Capita Food Supply (kcal/person/day)
2015	236.0	247.0	0.0	360990.0	6.3
2016	243.0	259.0	0.0	379665.0	6.5
2017	249.0	258.0	0.0	378272.1	6.4
2018	255.0	258.0	0.0	378818.3	6.3
2019	260.0	260.0	0.0	381654.5	6.3
2020	269.0	270.0	0.0	395600.3	6.5
2021	276.0	276.0	0.0	405572.8	6.6
2022	284.0	284.0	0.0	416385.7	6.7

### 3.2. Demographic status of the respondents

The survey reveals that most respondents were male, and the age distribution is well balanced. Most had at least secondary education, and common occupations included farming, service, and business. A significant portion lived in rural or semi-urban areas, and the majorities were Muslim. Monthly income and expenditure were mostly concentrated in the BDT 30,001–40,000 and BDT 20,001–30,000 ranges, respectively, indicating a modest but stable economic status among respondents (Table 3).

**Table 3.** Socioeconomic characteristics of the respondents in the study.

Item		Frequency	Percentage
Gender	Male	316	85.6
	Female	53	14.4
Age Group	< 25	10	2.7
	26–35	84	22.8
	36–45	91	24.7
	46–55	95	25.7
	> 56	89	24.1
Education	No education	13	3.5
	Up to primary	107	29.0
	Up to secondary	128	34.7
	Tertiary	121	32.8
Occupation	Farmer	109	29.5
	Businessman	83	22.5
	Service holder	96	26.0
	Housewife	20	5.4
	Others	61	16.5
Residence	Rural	178	48.2
	Semi-Urban	118	32.0
	Urban	73	19.8
Religion	Muslim	341	92.4
	Hindu	28	7.6
Monthly Income	< 20,000	5	1.4
	20,001–30,000	46	12.5
	30,001–40,000	182	49.3
	40,001–50,000	95	25.7
	50,001–60,000	26	7.0
	> 60,001	15	4.1
Monthly Expenditure	< 10,000	5	1.4
	10,001–20,000	111	30.1
	20,001–30,000	198	53.7
	30,001–40,000	40	10.8
	40,001–50,000	15	4.1
	> 50,001	0	0

**Figure 3.**
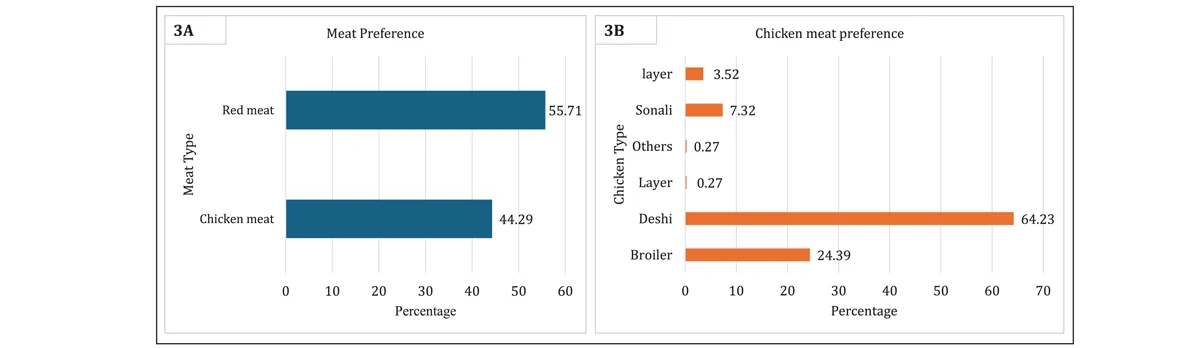
A) Meat consumption preferences among the surveyed population. B) Chicken meat consumption preferences among the surveyed population. 3A: Relative preference for red and white meat among respondents, reflecting dietary choice patterns within the study population. 3B: Preferences for different types of chicken meat, indicating consumer selection behavior and variability in poultry meat consumption.

From Figure 3A, it is indicated that the preference is almost the same for red meat (four-footed animal meat) and white meat (poultry meat). Among the respondents, about 64% prefer Deshi chicken, whereas about one-fourth of the respondents chose broiler compared to other chicken meats. Some people also prefer Sonali and Layer chickens (Figure 3B).

**Figure 4.**
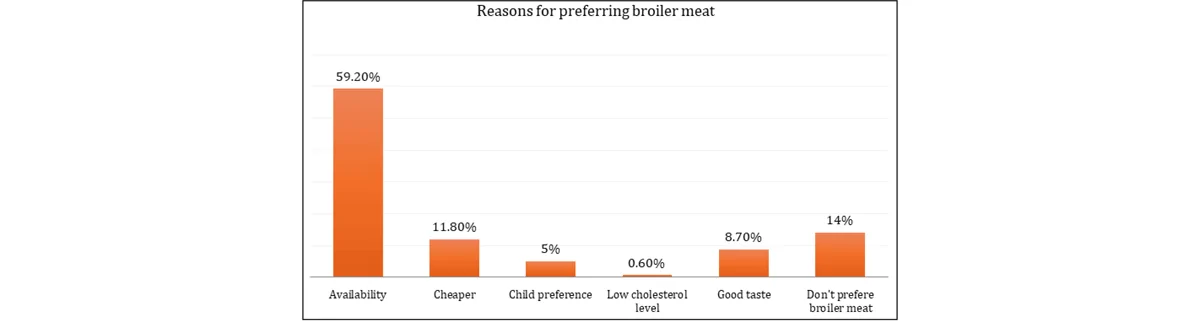
Reasons for preferring broiler meat among the surveyed population.

All the respondents were again asked why they eat broilers more than others, and they stated that the causes are availability (59.2%) and cheaper price (11.8%), followed by good taste (8.7%) and child preference (5%); and even 14% of people say they don’t like broilers, but still they eat them because they lack alternative protein sources (Figure 4).

Consumer perceptions of broiler meat were measured using a five-point Likert scale, with the responses ranked based on index values. A hierarchical ranking of pre-defined perception points revealed that the most common reason for rejecting broiler meat was the belief that broiler vendors do not maintain hygiene at processing points, and the second point is that broilers can spread different diseases. People also think that poultry meat contains unsafe chemicals, followed by using growth hormones. A significant number also believe that growth hormones (4.2) and heavy metals (4.1) are present in broiler meat and that broilers are less nutritious than desi meat (4.06). Other concerns include environmental pollution from heavy metals (3.97) and feed contamination (3.86). And lower-ranked statements are grouped, like broiler cause cancer (3.36), disabilities from childbirth (2.72), use of formalin (2.63), or related to coronavirus (2.47), which reflects respondents who mostly disagree and don’t believe that statement awareness with low perception risk (Table 4). The perception-based results serve as an important behavioral context for the regression outcomes, given that negative beliefs about broiler meat could indirectly affect consumption behaviors.

**Table 4.** Consumer perception ranking based on Likert scale responses.

Statement	Strongly agree	Agree	Disagree	Neutral	Strongly disagree	Likert score	Rank
Broiler vendors do not maintain hygiene at processing points	168 (45.5%)	197 (53.4%)	2 (0.5%)	1 (0.3%)	1 (0.3%)	4.43	1
Presence of antibiotic residue in broiler meat	112 (34.4%)	204 (63.6%)	0 (0%)	5 (1.2%)	0 (0%)	4.31	2
Broiler can spread different disease	132 (35.8%)	227 (64.5%)	5 (1.4%)	4 (1.1%)	1 (0.3%)	4.31	2
Various unsafe chemicals are used in broiler production	175 (47.4%)	127 (34.4%)	37 (10%)	29 (7.9%)	1 (0.3%)	4.29	3
Different growth hormones are used in broiler production	147 (39.8%)	185 (50.1%)	5 (1.4%)	30 (8.1%)	2 (0.5%)	4.2	4
Broiler meat contains heavy metals	100 (27.1%)	237 (64.2%)	1 (0.3%)	31 (8.4%)	0 (0%)	4.1	5
Broiler contains less nutrient per unit than Deshi meat	118 (32.0%)	186 (50.4%)	35 (9.5%)	30 (8.1%)	0 (0%)	4.06	6
Heavy metals of broiler production can pollute the environment	113 (30.6%)	162 (43.9%)	64 (17.3%)	30 (8.1%)	0 (0%)	3.97	7
Boiler feed contains heavy metals	86 (23.3%)	186 (50.4%)	59 (16%)	38 (10.3%)	0 (0%)	3.86	8
Unsafe feed is used in broiler production	104 (28.2%)	114 (30.9%)	91 (24.7%)	60 (16.3%)	0 (0%)	3.71	9
Conventional or regular broiler production is harmful for the environment	41 (11.1%)	205 (55.6%)	63 (17.1%)	60 (16.3%)	0 (0%)	3.61	10
Broiler meat consumption can develop cancer	19 (5.1%)	189 (51.2%)	66 (17.9%)	95 (25.7%)	0 (0%)	3.36	11
Unhygienic water is used in various steps of broiler processing	14 (3.8%)	80 (21.7%)	153 (41.5%)	121 (32.8%)	1 (0.3%)	2.95	12
Broiler meat consumption can cause disabled childbirth	17 (4.6%)	78 (21.1%)	121 (32.8%)	92 (24.9%)	61(16.5%)	2.72	13
Formalin is used in broiler production	21 (5.7%)	41 (11.1%)	152 (41.2%)	93 (25.2%)	62(16.8%)	2.63	14
Broiler can spread corona virus	3 (0.8%)	11 (3%)	212 (57.5%)	75 (20.3%)	68(18.4%)	2.47	15

An Ordinary Least Squares (OLS) method was conducted to examine the determinants of broiler meat consumption in Bangladesh. The dependent variable was the frequency of consumption per week, while key explanatory variables included place of residence, age, gender, total family members, education, occupational income, and expenditure. The results indicate that residence, education, occupation, and monthly income were statistically significant predictors of consumption (Table 5). Urban and semi-urban residents have higher rates of broiler meat consumption than rural residents, which is in agreement with the differences in availability and accessibility of processed products and changing dietary preferences specifically occurring around urban and semi-urban localities.

The positive effect of education: a one-unit shift between no level of education and secondary is marginally significant, whereas for tertiary education it is significant at the 5% level, indicating there was an increase by 0.95 units if education increased. Contrarily, broiler meat consumption had inversely significant relationships with occupational status. Significant inverse relations were observed in service holders and business professionals and monthly income. This is consistent with the results from the OLS regression, which found a negative association. Similar trends were found regarding household income: The likelihood of broiler meat consumption decreased as income increased. These results suggest a shift in preference among higher-income groups toward alternative protein sources perceived as safer, more diverse, or of higher quality.

Hence, socioeconomic status has a significant impact on dietary habits. Lower- and middle-income households are more reliant on broiler meat, primarily because of its affordability and widespread availability relative to other meats. In contrast, higher-income consumers show a tendency to reduce their intake of conventional broiler meat in favor of premium or certified products that assure quality and safety standards [14, 22]. Overall, the findings highlight a dual pattern: broiler meat continues to be an important protein source for the majority of Bangladeshi households, but rising income and occupational shifts are gradually altering consumption preferences toward diversified and higher-quality meat products.

The findings reveal that consumer attitudes toward broiler meat in Bangladesh are shaped more by perceptions of risk than by nutritional value. The strong agreement that vendors fail to maintain hygiene and that broiler meat contains chemicals, growth hormones, and heavy metals echoes earlier reports of widespread mistrust [18, 28]. Misconceptions such as the belief that broiler meat can cause cancer, although less strongly endorsed, illustrate how misinformation and fear can distort dietary behavior, even in the absence of scientific evidence [20]. These results suggest that consumer rejection is socially constructed through rumor, media narratives, and anecdotal accounts, which can have as much influence as economic factors in shaping demand [16].

The regression results further demonstrate the complex role of socioeconomic status in meat consumption. People who lived in cities or suburbs were more likely to eat broiler meat because they had access to supermarkets and packaged goods, which often mean better safety standards [21]. In contrast, income and occupational status exhibited a negative correlation with consumption, suggesting that affluent consumers are replacing conventional broiler chicken with Deshi chicken or certified products, a trend aligned with dietary transitions noted in other emerging economies [9, 14, 22]. Interestingly, education had a positive effect, which means that people who are educated value familiarity and low prices. This could be because broiler meat is used a lot in fast food, which is popular with younger and more educated people. A study involving university students indicated significant poultry meat consumption attributed to convenience and simplicity of preparation [29].

Taken together, these results suggest that Bangladesh’s broiler sector is caught between affordability-driven reliance among lower- and middle-income households and trust-driven disengagement among wealthier groups. If this pattern persists, the broiler meat will be stigmatized as a “poor man’s protein,” undermining its broader market sustainability. Addressing this requires not only strengthening hygiene and certification systems but also implementing awareness campaigns to communicate scientific evidence and rebuild consumer trust [14, 27]. Transparent labeling, stricter regulatory enforcement, and public–private partnerships could play pivotal roles in restoring confidence and ensuring that the poultry sector continues to contribute to food security in Bangladesh [6, 21]. Supporting this, evidence from the Mymensingh district shows that 64% of surveyed consumers prefer labeled broiler meat, while 71% favor certified products [29]. Moreover, consumers have demonstrated a positive willingness to pay (WTP) for safe broiler meat, with a contingent valuation study estimating a premium of approximately BDT 39.87 per kg [30].

### 3.3. Limitations

The use of proxy variables to represent behavioral constructs may not fully capture the underlying psychological dimensions of consumer decision-making in this study. Also, the cross-sectional nature of the data limits the ability to establish causal relationships. Future research may incorporate longitudinal data and direct measurement of behavioral constructs to provide deeper insights.

## 4. Conclusions

The research provides evidence that shows despite the rapid expansion of the poultry sector in Bangladesh and its continued role in providing a source of affordable protein, growth is hampered by some persistent consumer misconceptions. Broiler meat is relatively cheap and accessible, but consumer confidence is undermined by concerns about hygiene, disease risks, and undocumented use of chemicals, growth hormones, and heavy metals. The econometric results reveal further that socioeconomic factors such as residence, age, income, and occupational status shape consumption behavior, with lower- and middle-income households being more dependent on broiler meat, while higher-income groups increasingly are shifting to alternative products. Broiler meat continues to be essential in meeting protein needs for most Bangladeshi households; however, declining trust has an adverse effect on both smallholder sustainability and the poultry industries as a whole. Tackling this problem requires integrated strategies involving consumer education and transparent food safety and labeling systems, as well as stricter regulatory enforcement. Public–private partnerships and investment in sustainable, traceable, and environmentally responsible production systems are also vital for restoring confidence and protecting poultry’s continuing contribution to food security and nutrition. Overall, addressing consumer misconceptions and strengthening food safety governance are essential for sustaining the growth of the poultry sector. Improving transparency, certification systems, and public awareness will be critical to rebuilding consumer trust and ensuring the long-term contribution of broiler meat to food security and nutrition in Bangladesh.

## Data Availability

The data presented in this study are available from the corresponding author upon reasonable request.

## List of abbreviations

OLS, Ordinary Least Squares; *E. coli*, *Escherichia coli*; kg, kilogram; kcal, kilocalories.
